# Total arch distalization: A CBCT space assessment study

**DOI:** 10.6026/973206300223214

**Published:** 2026-05-31

**Authors:** Indulakshmi K.S, Gejo Johns1, Jose Sunny, Ajeesha Nair J.S, Arjun K, Renjith Madhavan

**Affiliations:** 1 Department of Orthodontics and Dentofacial Orthopaedics, Annoor Dental College and Hospital, Muvattupuzha, Ernakulam, Kerala, India; 2 Department of Periodontics Century Dental College, Kasaragod, Kerala, India

**Keywords:** CBCT, distalization, PAS, root morphology

## Abstract

Distalization of the maxillary and mandibular arches is a commonly performed space-gaining procedure used to avoid extractions. However, successful total arch distalization requires careful assessment of several factors. Therefore, it is of interest to evaluate key parameters for planning total arch distalization in the maxilla and mandible using cone-beam computed tomography, including posterior available space and root morphology. CBCT of maxilla and mandible taken for various purposes where used. The views required are sagittal and axial sections. CBCT images were saved as DICOM files to generate quantitative measurements. The PAS in the maxilla region is higher compared to that in the mandibular region with a statistically significant difference.

## Background:

Molar distalization is a conservative, non-extraction orthodontic approach used in the treatment of Class II and Class III molar relationships in conjunctions with dental crowding relieves, while avoiding excessive arch expansion that might affect facial esthetics and long-term occlusal stability [1]. Posterior available space (PAS) can differ between males and females due to anatomical and developmental variations in dental arch morphology, growth patterns, and hormonal influences [2]. These differences are essential considerations in orthodontic treatment planning, particularly when evaluating the feasibility of distalizing teeth in cases such as Class II malocclusions or dental crowding. Evaluating the posterior alveolar space (PAS) in the retromolar region of the mandible and the maxillary tuberosity (MT) area is essential before commencing molar distalization or uprighting. A thorough assessment minimizes the risk of periodontal complications and contributes to achieving precise and stable treatment outcomes [3]. Therefore, it is of interest to the parameters on maxilla and mandible using CBCT.

## Materials and Methods:

This *in vitro* investigation was carried out in the Department of Orthodontics at Thai Moogambigai Dental College and Hospital, Chennai, India. Ethical clearance for the study was granted by Institutional Review Board and the Institutional Ethics Committee on 02.06.2023 (Approval No: 402/2023/IEC/TMDCH Dt.02.06.2023). Comprehensive details regarding the study objectives, anticipated duration, and research procedures were provided to all participants, and informed consent was duly obtained prior to participation.

## Inclusion criteria:

[1] Class I, II and III skeletal malocclusion in patients between 18-30 years

[2] Level of the alveolar bone located superior to the furcation area of the mandibular molars.

[3] CBCT images of good quality

[4] Presence of permanent 2nd molars

## Exclusion criteria:

[1] Ectopic eruption of 2nd molars

[2] Previous history of orthodontic treatment

[3] Craniofacial syndromes

[4] Congenitally missing teeth

[5] CBCT images with inadequate coverage & insufficient quality due to artifacts

## Procedures:

40 patients (20 men and 20 women) between the age group 18-30 years who had taken CBCT of maxilla and mandible for various purposes where taken for the study. The views required are sagittal and axial sections. CBCT images were saved as DICOM files to generate quantitative measurements. Carestream imaging software was used in the study for viewing and analysing the CBCT. Posterior available space the posterior available space (PAS) was determined using axial and sagittal views obtained from CBCT scans of the maxilla and mandible. Measurements in the maxilla were recorded from the furcation level of the second molar's distobuccal root to the outer cortical border of the tuberosity. In the mandible, measurements extended from the furcation of the distal molar root to the outer cortical surface of the retromolar region. The measurements in the axial and sagital section for maxilla and mandible are obtained and the mean is calculated to obtain the posterior available space for each patient.

## Root morphology of 2nd molar:

This parameter was assessed using CBCT. Based on the morphology observed in axial sections, the mandibular second molar roots were classified into three types: single-root, double-root and C-shaped root configurations.

## Results and Discussion:

Statistical analyses were conducted using IBM SPSS Statistics version 26.0. Descriptive data are presented as mean, range, minimum, maximum, and standard deviation. Normality was assessed using the Shapiro-Wilk test, confirming a normal distribution. Accordingly, Continuous variables were analyzed using independent samples t-tests, whereas categorical variables were evaluated with Chi-square tests and Spearman's rank correlation. Statistical significance was set at p < 0.05. Table 1 shows the mean, standard deviation, minimum, maximum, and range of PAS in the maxilla and mandible, compared between male and female subjects. From the above table, the highest posterior available space in the maxilla is observed in males (8.93 ± 0.7mm) compared to females (7.76 ± 0.9mm). Similarly, in the mandible, the highest posterior available space is found in the males (7.93 ± 0.7mm) compared to females (6.76 ± 0.9mm). It is thereby inferred that the PAS is higher in males for both the maxilla and mandible region. On comparing the overall posterior available space between the maxilla and mandible, a statistically significant difference is observed with a significance value i.e., p-value = 0.03 (Table 2). The PAS in the maxilla region is higher (8.84 ± 2.5mm) compared to that in the mandibular region (6.76 ± 5.7 mm) with a statistically significantt difference. In Table 3, the root morphology for maxilla having the single pattern and double pattern, the highest frequency is observed in females with 65% and 25%, respectively. While C-shaped root morphology has the highest frequency with 25% in the males. On comparing the root morphology for maxilla, there is no considerable difference between genders. In Table 4, the root morphology for mandible having the single pattern, highest frequency is observed in females with 55%. While double pattern and C-shaped root morphology has the highest frequency in males with 15% and 40%, respectively. On comparing the root morphology for mandible, there is an observable difference between the 2 genders. (p=0.01). Table 5 comparing the root morphology between the maxilla and mandible, a significant difference (p=0.04) is observed between the 3 groups with the highest value observed in mandible for C-shaped root morphology.

In Table 6, a positive correlation is observed between the posterior available space and the C shaped canal in the mandible region with a significant value of 0.004. This concludes that as the presence of C shaped canal increases, the posterior available space also increases or vice versa. Modern orthodontic approaches increasingly prioritize conservative, non-extraction methods. One such method for addressing Class II malocclusion is upper molar distalization, which aims to establish a Class I molar relationship without the need for extractions. While traditional distalization techniques often depend on patient cooperation with appliances like headgear or elastics, newer intraoral devices have been developed to reduce this dependency. These appliances are capable of moving the upper molars distally with minimal to no reliance on patient compliance [4]. Distalization is typically performed before the retraction of the anterior teeth. It is most effective when initiated before the premolars upper second molars erupt, as this timing allows for optimal use of the leeway space [5]. This research seeks to evaluate the posterior available space (PAS) for molar distalization and to examine additional anatomical factors within the maxilla and mandible that influence distalization such as root morphology of the second molar. Previous research by Park *et al.* [6] Kim *et al.* [7], and Chen *et al.* [8] emphasized the importance of assessing PAS in the retromolar and tuberosity (MT) areas of the mandible and maxillary areas prior to molar distalization or uprighting. Such assessments are crucial to avoid periodontal complications and to ensure favourable treatment outcomes. In the present study, alongside the evaluation of PAS, and root canal morphology were also analyzed for their relationship with available space. PAS was measured using CBCT and compared between the maxilla and mandible, as well as between genders. Findings revealed that the maxilla had a greater average PAS (8.84 ± 2.5 mm) compared to the mandible (6.76 ± 5.7 mm). Gender-wise analysis indicated that males exhibited more available space in both arches (8.93 ± 0.7 mm) than females (7.76 ± 0.9 mm). These results are similar to with those of Kim *et al.* [9] and Kim *et al.* [10], who highlighted the maxillary tuberosity as a region with sufficient bone volume for safe molar distalization. Recent CBCT investigations have indicated that the posterior anatomical boundary of the mandibular PAS at the tip of the root coincides with the cortical plate of the lingual mandible. This observation implies that conventional radiographic methods may overestimate the actual mandibular PAS, highlighting the necessity of CBCT for accurate evaluation and treatment planning in cases involving considerable molar distalization C-shaped root canal morphology was found to be more common in males in both arches. Between the two arches, the mandible demonstrated a higher incidence of C-shaped canals. These observations align with the findings of Takuma Funakoshi *et al.* [11] who classified canal morphology into single, double, and C-shaped patterns. A significant positive correlation (p = 0.004) was found between the occurrence of C-shaped root morphology and an increase in posterior available space (PAS). This observation supports the findings of Keiichiro Iguchi [12], which suggested that the posterior anatomic limit of the mandible (MPAL) was notably larger in cases where the second molar of the mandibular arch exhibited a C-shaped root. Their study suggested that this root configuration could contribute to greater space availability for molar distalization in the mandible.

Clinically, total arch distalization can unintentionally cause mandibular arch expansion, which may result in a buccal crossbite, particularly in skeletal Class III patients who commonly exhibit underlying transverse discrepancies [13]. Despite these findings, this study has certain constraints. The size was limited, and variations in skeletal relationships and malocclusion types were not included. Future research with a larger and more diverse sample population is recommended to strengthen these results. Additionally, examining the influence of erupted third molars on distalization potential could yield further insights. The application of CBCT in subsequent studies may also enhance the precision of angulation and spatial evaluations. Elghawy *et al.* in his study suggested the use of intraoral (IO) or extraoral (EO) appliance for distalisation. The use of intraoral appliance revealed an improvement in the airway volume (AWV) and minimum constricted area (MCA) to a modest degree [14]. A study conducted by Hong *et al.* indicates that in the long term, developing mandibular third-molar buds shifted downward and buccally following total arch distalization. These results indicate that clinicians should be aware of the increased risk of third-molar impaction in adolescents undergoing mandibular total arch distalization [15]. Owayda *et al.* in his study suggested that Total maxillary arch distalisation is effective in camouflaging class II division 1 malocclusion [16].

## Conclusion:

PAS was found to be greater in the maxilla. Male subjects exhibited higher PAS values in both arches. The occurrence of C-shaped root morphology was most frequent in males in both the arches, with mandibular arch showing greatest overall prevalence. Furthermore, an increase in the presence of C-shaped canals corresponded with an increase in posterior available space, indicating a positive association between the two variables.

## Clinical significance:

This study provides essential CBCT-based insights for orthodontic treatment planning by quantifying posterior available space and root morphology. Understanding these variations between arches and genders enables safer, more precise total arch distalization, minimizing risks of root contact or periodontal damage and enhancing treatment predictability and stability.

## Figures and Tables

**Table 1:** Descriptive statistics for posterior available space for both maxilla and mandible between two genders

	**Maxilla**		**Mandible**	
	**Male**	**Female**	**Male**	**Female**
Mean ± SD	8.93 ± 0.7	7.76 ± 0.9	7.93 ± 0.7	6.76 ± 0.9
Minimum value	6.7	5.66	6.7	5.66
Maximum value	9.38	9.13	9.38	9.13
Range	2.68	3.47	2.68	3.47

**Table 2:** Comparing the available posterior space for both the maxilla and mandible using unpaired t-tests

**Maxilla**	**Mandible**	**t- value**	**p-value**
**Mean ± SD**	**Mean ± SD**		
(N=40)	(N=40)		
8.84 ± 2.5	6.94 ± 5.7	3.18	0.03*
*Significant level p < 0.05.

**Table 3:** Comparing the root morphology for mandible between two genders using Chi-square test.

**Root**	**Maxilla**		**Chi-square**
**Morphology**	**Frequency %**	**Frequency %**	**value**
	Males (N=20)	Females (N=20)	
Single pattern	12 (60%)	13 (65%)	
Double pattern	3 (15%)	5 (25%)	0.47
C shaped	5 (25%)	2 (10%)	
*Significant level
p < 0.05.

**Table 4:** Comparing the root morphology for mandible between two genders using Chi-square test.

**Root**	**Mandible**		
Morphology	Frequency %	Frequency %	Chi-square value
	Males (N=20)	Females(N=20)	
Single pattern	9 (45%)	11 (55%)	
Double pattern	3 (15%)	2 (10%)	0.01*
C shaped	8 (40%)	7 (35%)	
*Significant level
p < 0.05.

**Table 5:** Comparing the root morphology for both maxilla and mandible using Chi-square test.

**Root**	**Maxilla**	**Mandible**	**p-value**
Morphology	(N=40)	(N=40)	
Single pattern	25 (62.5%)	20 (50%)	0.04*
Double pattern	10 (25%)	5 (12.5%)	
C shaped	5 (12.5%)	15 (37.5%)	
*Significant level
p < 0.05.

**Table 6:** Correlation between the posterior available space and C shaped canal for mandible using Spearman's rank correlation test.

	**Posterior available space Mandible(N=40) Correlation coefficient**	**Posterior available space Mandible(N=40) Sig. (2-tailed)**
C shaped canal (Mandible)	0.669	0.04*
